# Tumor markers of bladder cancer: the schistosomal bladder tumors versus non-schistosomal bladder tumors

**DOI:** 10.1186/1756-9966-28-27

**Published:** 2009-02-25

**Authors:** Ahmed S Abdulamir, Rand R Hafidh, Haider S Kadhim, Fatimah Abubakar

**Affiliations:** 1 Microbiology Research Department, University Putra Malaysia, 43400, UPM, Serdang, Malaysia; 2 Institute of Bioscience, University Putra Malaysia, 43400, UPM, Serdang, Selangor Darul Ehsan, Malaysia

## Abstract

**Background:**

The aim of this study is to comparatively elucidate the underlying molecular pathways and clinicopathological criteria in schistosomal bladder tumor (SBT) versus non-schistosomal bladder tumor (NSBT).

**Methods:**

This study explored the role of p53, p16, bcl-2, ki-67, c-myc, Rb and EGFR, by using Immunohistochemistry assay, in 45 SBT and 39 NSBT patients in comparison with 16 schistosomal chronic cystitis (SC), 28 non-schistosomal chronic cystitis (NSC), and 20 normal control (CTL) subjects. The studied markers in SBT and NSBT were correlated with different clinicopathological criteria namely, tumor histopathology, grading, invasiveness, stage, and presentation of the disease.

**Results:**

SBT was associated with high grade invasive squamous cell carcinoma (SCC) while NSBT was associated with lower grade less invasive transitional cell carcinoma (TCC). The expression of p53, bcl-2, c-myc, and EGFR was higher in SBT than in NSBT while Rb was higher in NSBT than in SBT. However, p16 and ki-67 were not different between SBT and NSBT. The profile of molecular markers in SC was similar to NSC except for EGFR which was higher in SC than in NSC. Both SC and NSC showed higher level of p53, bcl-2, ki-67, and EGFR than in CTL group while p16, Rb, and c-myc were not different. p53 was associated with high grade SCC in both SBT and NSBT. Bcl-2 was associated with high grade invasive tumors in SBT and NSBT. P16 was associated with low grade, late stage, and recurrent SBT and high grade, invasive, late stage, and recurrent NSBT. Rb was associated with SCC in SBT, invasive tumors in NSBT, and late stage and recurrent presentation in both SBT and NSBT. C-myc was associated with high grade, invasive, and late stage SBT and SCC, high grade, invasive, and late stage NSBT. EGFR was associated with invasive SCC in SBT and invasive, high grade, and late stage TCC in NSBT. ki-67 was associated with invasive SBT and high grade late stage NSBT.

**Conclusion:**

SBT and NSBT showed distinct molecular profile of tumor development and progression which can be taken into consideration in fine adjusting the anti-cancer therapy for SBT and NSBT.

## Introduction

Bladder cancer is the second most common malignancy of the genitourinary system in both males and females [1]. The most common type diagnosed in North America, South America, Europe, and Asia is transitional cell carcinoma (TCC), which is mainly non-schistosomal bladder tumors (NSBT), followed by squamous cell carcinoma (SCC) which is found more in geographical regions where schistosomiasis is prevalent [1]. The neoplastic changes in the urothelium of bladder is a multistep phenomenon [2]. The exact genetic events leading to urothelial transformation involve the activation of oncogenes, inactivation or loss of tumor suppressor genes, and alterations in the apoptotic gene products [3].

One of the conditions leads to bladder cancer in Africa, the Middle East, and Asia is schistosomiasis [4,5]. *S*. haematobium is the most predominant species in the Middle East, Asia, and Africa and the most implicated in the schistosomal bladder tumors (SBT) in these regions [6,7].

C-myc is implicated in bladder cancer, the genetic mechanism causing overexpression of the *c-myc *gene in bladder cancer is unknown. It could be related to hypomethylation [8] and its overexpression has been shown to be associated with high-grade bladder cancer [9]. Another oncogene implicated in bladder cancer, namely epidermal growth factor receptor (EGFR). Overexpression of EGFR has been described in several solid tumors including bladder, breast, colorectal, prostate, and ovarian cancers [10]. And 70% of muscle-invasive bladder cancers express EGFR, which is associated with poor prognosis [11].

The majority of aggressive and invasive bladder carcinomas have alterations in the tumor suppressor genes products such as retinoblastoma (Rb) [12]. A study revealed that tumor expression of Rb proteins in locally advanced bladder cancers was found abnormal [13]. Another tumor suppressor protein, p53, plays a vital role in the regulation of cell cycle. The defective p53 in human cancer leads to the loss of p53-dependent apoptosis, proliferative advantage, genomic instability and DNA repair and angiogenic control loss [14]. Mutations in the p53 gene result in the production of dysfunctional protein product with a prolonged half-life compared to the wild-type protein [14]. On the other hand, p16, which is a tumor suppressor protein, was found almost abnormal in the advanced bladder cancers where it was severely lowered and impaired in function. [12].

Overexpression of bcl-2 has been reported in a wide variety of cancers including prostate, colorectal, lung, renal, bladder and leukemia [15]. Several studies have provided conclusive evidence that elevations in bcl-2 expression cause resistance to chemotherapy and radiotherapy and increases the proliferation [16]. On the other hand, Ki 67 is used to evaluate the proliferative potential of any tumor as it is one of the important markers for cell proliferation [17].

There was no previous study explored the profiling of molecular markers in SBT and NSBT with respect to tumor suppressor proteins: p53, Rb, and p16, oncogenes: c-myc, and EGFR, an antiapoptotic protein: bcl-2, and a proliferative protein, ki-67 together in one study. Moreover, c-myc, EGFR, and Rb have not been investigated thoroughly in relation to SBT. In addition, this study compared the profile of the studied markers among SBT, NSBT, chronic schistosomal cystitis (SC), chronic non-schistosomal cystitis (NSC), and normal control subjects (CTL). This study is believed to highlight the essential molecular targets that can be important candidates for anti-cancer therapy in both SBT and NSBT.

## Materials and methods

### The population of the study

#### Bladder cancer patients

Eighty four (84) patients (63 men and 21 women) with bladder cancer, confirmed by histopathology, were included in this study in the period from March 2007 to May 2008. The patients with bladder cancer were retrieved, examined, interviewed, and sampled in the region of The Middle East (Jordan, Syria and Iraq). The investigational study was conducted in the University Putra Malaysia (UPM) in Malaysia. The patients' age ranged 38–72 years old with mean age 59.49 ± 5.7 years. The involved patients were selected from 3 central teaching hospitals without bias to age, sex, or cancer pathology. The involved patients were sampled before the beginning of anti-cancer therapy. The diagnosis of bladder cancer was established by doing urine cytology and diagnostic cyctoscopy where the histopathology of biopsies confirmed the diagnosis of bladder cancer and determined the tumor grade, local invasiveness, and the histopathological type of cancer. The tumor spread and metastasis was assessed by CT scans and cystoscopy.

Moreover, past schistosomal infection was monitored by retrieving the previous medical records. The current diagnosis of schistosomiasis was done by cystoscopy through finding bilharzial granuloma or egg in histopathological sections. Accordingly, patients with bladder cancer were categorized into 45 patients with SBT and 39 patients with NSBT. The involved patients with bladder cancer did not show extra-bladder tumors. The stages of the retrieved patients ranged from I to IV.

Moreover, cancer patients were categorized accordingly into muscle invasive (T2, T3, and T4) and non invasive tumors (Ta, T1, and CIS). For comparative purposes with previous reports, the 1973 WHO grading system (papilloma, G1, G2 or G3) was used in this study which is still the most commonly used system despite being superseded by the 2004 WHO. The retrieved tumors were histologically categorized as low grade (grades 1–2) and high grade (grade 3). Moreover, the tumor morphology was categorized by cystoscopy into 71 cases papillary, 12 cases sessile and 1 case nodular. Written consents were granted by the involved subjects for sampling. The handling with human subjects was done under the permission of the regional committee of Ethics for biomedical research.

#### The group of benign bladder lesions

This group encompassed 44 untreated cases of chronic cystitis patients (29 men and 15 women) with mean age 57.62 ± 3.78 years. The patients with chronic cystitis were retrieved and sampled from the same geographical region of cancer patients, the Middle East. Patients with chronic cystitis were diagnosed by urine cytology and diagnostic cystoscopy coupled with histopathological examination. There were no signs of premalignant lesions (squamous metaplasia, dysplastic changes, or leukoplakia) nor were signs of prostatic enlargement found. Under the same diagnostic protocols done for bladder cancer patients, the chronic cystitis patients were grouped into 16 schistosomal cystitis (SC) patients and 28 non-schistosomal cystitis (NSC) patients.

#### Control group

Twenty age- and sex- matched individuals (12 men and 8 women) at mean age 58.3 ± 6.1 years old were involved from the Middle East region. Their bladders were investigated by routine cystoscopy and biopsies were taken. They were found free of bladder cancer or any other bladder disease or inflammation, therefore, they were considered as control group (CTL).

### Processing of biopsies

The bladder cancer patients, the chronic cystitis patients, and CTL subjects underwent transurethral resection of bladder tumor (TUR-BT), cystitis tissues, and normal mucosal tissues respectively. The retrieved specimens were composed of multiple pieces, 2–5 mm in thickness. Specimens were immersed in 10% formalin in order to make a paraffin block. The histopathological paraffin blocks of biopsies were sectioned into 4 um thick sections. Hematoxylin and Eosin slides were prepared and examined by a histopathologist for confirming the histopathological diagnosis, the grade, and the invasiveness of the tumor. A set of steps were pursued under the supervision of a pathologist to minimize as could as possible the fixation-related loss of target proteins. These steps were: minimal prefixation time of 1 hour, the use of cold 4% paraformaldehyde and cold fixation at 4°C, and short duration of fixation up to 5 hours [18]. Moreover, the paraffin-embedded sections processed for immunohistochemistry (IHC) assay were examined in a period not more than 3 days. It was stated that insignificant loss of nucleic acids or proteins was observed within the first 3 days of fixation-paraffin embedding [18].

### Immunohistochemistry assay

#### Antibodies

IHC staining was conducted using a set of mouse monoclonal antibodies; anti-p53, anti-p16, anti bcl-2, and anti-c-myc (InnoGenex, USA) and anti Ki-67, anti-Rb-1, and anti-EGFR (DakoCytomation). Secondary biotinylated goat anti-mouse antibodies were used (DakoCytomation). Antibodies were diluted in the recommended antibody diluting buffer (Dako). The working dilutions and the final concentrations of the primary antibodies were 1:100 and 0.005 mg/mL for anti-p53, 1:120 and 0.008 mg/mL for anti-p16, 1:75 and 0.006 mg/mL for anti-bcl-2, 1:100 and 0.01 mg/mL for c-myc, 1: 50 and 0.01 mg/mL for anti-Rb-1, 1:200 and 0.005 mg/mL for anti-ki67, and 1:120 and 0.008 mg/mL for anti-EGFR antibodies. The used dilution and concentration of the biotinylated goat anti-mouse antibodies were 1:800 at final concentration 0.0025 mg/mL.

#### Immunohistochemistry procedure

The procedure of IHC was conducted according to the manufacturer instructions (LSAB2 Universal Dakocytomation strepavidin-biotin detection system). After baking slides in oven at 65°C overnight, slides were deparaffinized by applying sequential immersion for 5 min in xylene, 95% ethanol, 70% ethanol, and distilled water (DW). Autoclave-based antigen retrieval was standardized for each target protein. Slides were placed in a jar containing antigen retrieval solution (0.1 M citrate buffer from BDH at pH 6) and left in autoclave, for 0.5–8 min (variable time for each target protein) at 121°C. 100 μL of the diluted primary antibodies were then applied onto the sections and the slides were incubated in a humid chamber overnight at 4°C.

The next day, slides were rinsed gently with PBS (Merck)-Tween (Sigma) and placed in fresh PBS-Tween bath for 1 min. One-two drops of the diluted biotinylated secondary goat anti-mouse antibodies (DakoCytomation) were applied onto the sections and the slides were incubated in a humid chamber for 1 h at 37°C. After rinsing step, One-two drops of streptavidin-Horseradish peroxidase reagent (DakoCytomation) was applied onto the sections, slides were incubated for 30 min at 37°C. The prepared DAB-substrate chromogen solution was applied onto sections, Slides were incubated in dark at room temperature for 20 min. Mayer's hematoxylin stain was used as counterstain, then slides were dehydrated and mounted with DPX mounting fluid. In every run, two negative controls were used. The first negative control was antibody diluting buffer added alone without primary antibodies. This is essential for measuring the non-specific noise of staining. The second negative control was a known normal urothelium section devoid of any positive staining of the corresponding target molecule. On the other hand, a strong and consistently stained section was used as a positive control for each target. The detected staining noise, if any, was subtracted from the corresponding test section.

#### Staining analysis

**Figure 1:**
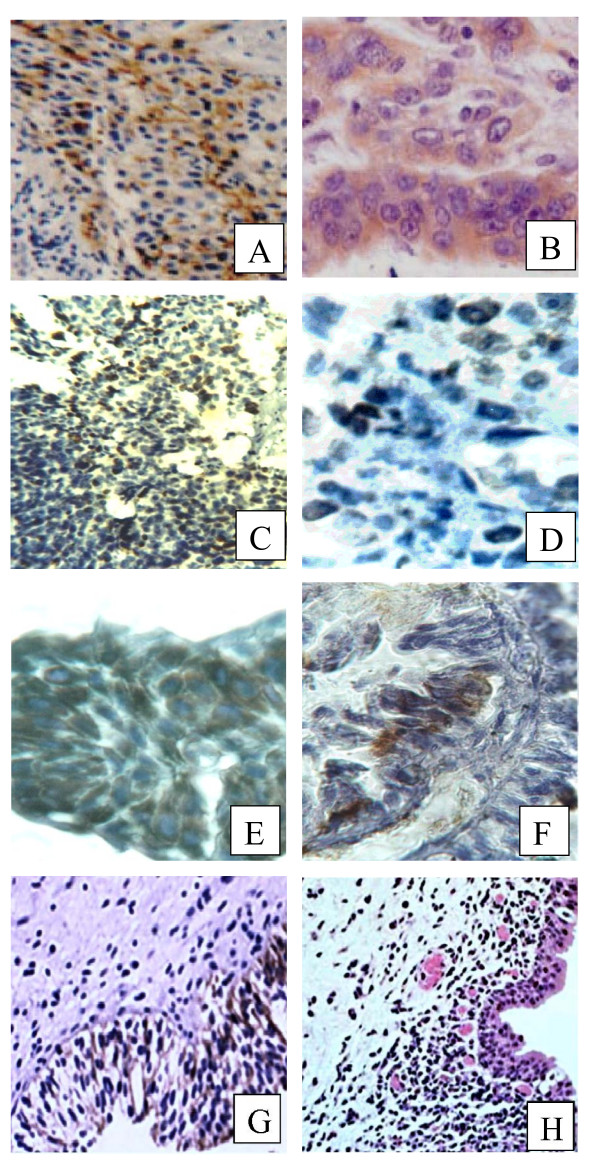
**Immunohistochemical staining of bladder tumor sections**. Immunostaining by peroxidase/DAB (brown) counterstained with hematoxylin. (A) SCC SBT, c-myc protein cytoplasmic staining in high grade tumor (X400). (B) SCC SBT, EGFR cytoplasmic staining in high grade tumor (X1000). (C) TCC NSBT, bcl-2 nuclear staining high-grade tumor (X400). (D) TCC NSBT, Rb nuclear staining in invasive tumor (X1000). (E) TCC NSBT, ki-67 protein cytoplasmic staining in high-grade tumor (X400). (F) TCC SBT, p53 nuclear stains in low-grade tumor (X1000). (G) SC, p16 nuclear staining (X400). (H) NSC, c-myc cytoplasmic staining (X200).

The tumor cell staining, membranous, cytoplasmic, and nuclear compartments were taken into consideration. Furthermore, staining of the stromal cells dispersed between tumor epithelial cells (not more than 5% of the total cells in the section) was taken into account as these cells reflected the same mutational abnormality of the epithelial cells. However, other stromal cells scattered throughout the section were not taken into account. The pattern of staining was dominantly nuclear for p53, p16, Rb, and bcl-2, nuclear and cytoplasmic for ki-67, cytoplasmic and membranous for EGFR, and mainly cytoplasmic for c-myc. Since differences in the staining intensity of the studied proteins were slight, the qualitative positive/negative system was used. The immunostained cells at moderate to intense dark brown color were considered positive while other cells were considered negative (Figure. 1). Under light microscope, the mean percentage of the positively stained cells in each section was calculated from 3 dense, medium, and light staining areas. In each area, the percentage of brown stained cells was calculated out of total countable cells in 5 high power fields. Due to the numerous, sometimes contradicting, scoring systems of the target proteins, the mean percentage of the positively stained cells was quantitively compared among the different groups of this study. To keep the scientific fidelity and to ensure the impartial evaluation, the immunostained slides were examined blindly by two scientists, one from the research team and a consultant histopathologist outside the research team.

### Statistical Analysis

Statistical analysis was conducted using SPSS software version 10 and MS Excel 2000. Chi-square test of independence was used for evaluating the significant association of histopathology type, tumor grade, tumor invasiveness, disease staging, and disease recurrence with SBT and NSBT groups. After proving that the studied groups obey the normal distribution pattern by using Kolmogorov and Semirnov normalization tests, parametric tests were used. Accordingly, student *t *test was used to measure the significant difference of the mean percentage of the positively stained cells for p53, p16, Rb, bcl-2, ki-67, c-myc, and EGFR proteins among the different groups of the study. Moreover, Pearson's correlation coefficient (r) was used to measure the correlating behavior of the studied markers with each other. P value less than 0.05 was considered as significant.

## Results

### Demographic features of the bladder cancer and cystitis patients

The demographic features of the involved patients with bladder cancer and chronic cystitis are summarized in (Table 1). It was found that the mean age of SBT and SC were less than of NSBT and NSC receptively (P < 0.05). Male: female ratio was higher in SBT and SC than in NSBT and NSC respectively (P < 0.05). On the other hand, there was no significant difference between bladder cancer, as a whole, and cystitis patients regarding mean age and sex ratio (P > 0.05). Moreover, there was no significant difference in age and sex ratio in relation to tumor histopathology, disease stage and presentation, tumor grade, tumor invasion, or the tumor growth pattern in both SBT and NSBT groups (P > 0.05).

**Table 1:** The demographic features of the involved patients with baldder cancer and chronic cystitis.

**Demographic feature**	**Value**
Bladder cancer	
Number of patients:	
SBT	45 (53.57%)
NSBT	39 (46.43%)
	
Sex of patients:	
SBT	38 men and 7 women
NSBT	25 men and 14 women
	
Recurrence of bladder cancer:	
First presentation	61 (72.62%)
Recurrent	23 (27.38%)
	
Age of patients:	
SBT	Range: 38–64 years, mean: 51.4 ± 6.2 years
NSBT	Range: 46–72, mean: 66.5 ± 5.3 years
	
Type of tumor growth:	
SBT	37 papillary 8 sessile
NSBT	34 papillary 4 sessile 1 nodular
	
Tumor muscle invasiveness:	
Invasive (T2, T3, and T4)	62 (73.81%) patients
Non invasive (Ta, T1, and CIS)	22 (26.19%) patients
	
Grading:	
Low grade (grade 1 and 2)	35 (41.66%) patients
High grade (grade 3)	49 (58.33%) patients
	
Histopathology of bladder tumors:	
SCC	52 (61.91%) patients
TCC	32 (38.09%) patients
	
Staging of bladder cancer patients:	
Stage I	9 (10.71%)
Stage II	13 (15.47%)
Stage III	18 (21.42%)
Stage IV	44 (52.38%)
Chronic cystitis	

Number of patients:	
SC	16 (36.36%)
NSC	28 cases (63.64%)
	
Sex of patients:	
SC	14 men and 2 women
NSC	15 men and 13 women
	
Age of patients:	
SC	mean age 62.5 ± 3.5 years
NSC	mean age 53.4 ± 4.2 years

### Molecular profile among SBT, NSBT, SC, NSC, and CTL groups

The immunostaining of the paraffin-embedded sections in terms of mean percentage of the positively stained cells for p53, p16, bcl-2, ki-67, Rb, c-myc, and EGFR proteins was compared among SBT, NSBT, SC, NSC, and CTL groups. It was shown that the molecular profiles of SBT and NSBT were different from each other and from that of SC, NSC and CTL groups.

**Figure 2:**
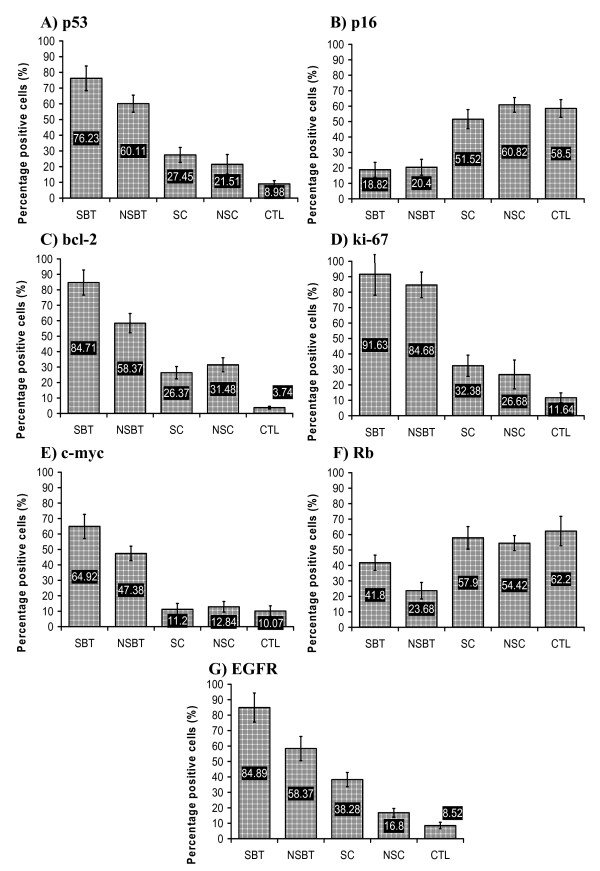
**The mean percentage of the positively immunostained cells for (A) p53, (B) p16, (C) bcl-2, (D) ki-67, (E) c-myc, (F) Rb, (G) EGFR in bladder tissue sections of SBT, NSBT, SC, NSC, and CTL groups**.

The mean percentage of the positively stained cells for p53 protein was higher in SBT than in NSBT (P < 0.05) and both SBT and NSBT showed higher p53 expression than in SC and NSC groups (P < 0.05) which both showed close levels of p53 expression (P > 0.05). However, SC and NSC showed higher levels of p53 than in CTL group (P < 0.05) (Figure. 2-A). P16 level of expression was almost similar among CTL, SC, and NSC groups (P > 0.05) while its level sharply decreased in both SBT and NSBT (P < 0.05) without any difference between SBT and NSBT (P > 0.05) (Figure. 2-B). Bcl-2 level of expression was higher in SBT than in NSBT (P < 0.05) and both showed higher bcl-2 expression than in SC and NSC (P < 0.05). The bcl-2 level was not different between SC and NSC (P > 0.05) which both showed higher expression than in CTL group (P < 0.05) (Figure. 2-C). Ki-67 expression was increasing from CTL towards SC and NSC (P < 0.05) and from SC and NSC towards SBT and NSBT (P < 0.05) without any significant difference between SC and NSC or between SBT and NSBT (P > 0.05) (Figure. 2-D). The level of c-myc in both SC and NSC was not higher than in CTL group (P > 0.05) but it was remarkably higher in SBT and NSBT than other groups (P < 0.05). Interestingly, c-myc was higher in SBT than in NSBT (P < 0.05) (Figure. 2-E). The expression of Rb was diminished in both SBT and NSBT when compared with CTL, SC, and NSC groups (P < 0.05). NSBT group showed lower Rb expression than in SBT group (P < 0.05). Rb level in both SC and NSC was close and not significantly different from that in CTL group (P > 0.05) (Figure. 2-F). The expression level of EGFR increased significantly from CTL group towards NSC, SC, NSBT, and SBT (P < 0.05) (Figure. 2-G).

### The clinicopthological features in SBT versus NSBT

The clinicopathological criteria in SBT and NSBT groups were compared with each other using chi square test for independence. It was found that SBT was associated with SCC rather than TCC, high grade tumors rather than low grade, and invasive tumors rather than non-invasive tumors (P < 0.05). On the other hand, NSBT was associated with TCC rather than SCC, lower grade tumors rather than high grade, and non-invasive rather than invasive tumors (P < 0.05). However, there was no association between SBT or NSBT and disease staging or presentation (P > 0.05) (Table 2). Moreover, there was no association between SBT or NSBT and the growth pattern of tumors (data not shown).

**Table 2:** The clinicopathological criteria in SBT versus NSBT

**Criteria (N)**	**SBT (45)** **N (%)**	**NSBT (39)** **N (%)**	**P value**
Histopathology			
SCC (52)	43 (82.69)	9 (17.3)	< 0.05
TCC (32)	2 (6.25)	30 (3.75)	
Tumor grade			
High grade (49)	33 (67.34)	16 (32.65)	< 0.05
Low grade (35)	12 (34.28)	23 (65.71)	
Tumor invasiveness			
Invasive (62)	38 (61.29)	24 (38.7)	< 0.05
Non-invasive (22)	7 (31.81)	15 (68.18)	
Tumor staging			
Late stage (III and IV) (62)	31 (50)	31 (50)	> 0.05
Early stage (I and II) (22)	14 (63.63)	8 (36.36)	
Presentation			
First presentation (61)	32 (52.45)	29 (47.54)	> 0.05
Recurrent (23)	13 (56.52)	10 (43.47)	

### The molecular profile of SBT and NSBT in regard to clinicopathological criteria

The mean percentages of the positively stained cells for p53, p16, bcl-2, ki-67, c-myc, Rb, and EGFR proteins were calculated with respect to the clinicopathological criteria of SBT and NSBT. This served to understand the behavior of the studied tumor suppressor proteins, oncogenes, proliferative and apoptotic markers in relation to histopathology, grade, invasiveness, disease staging, and presentation.

**Figure 3:**
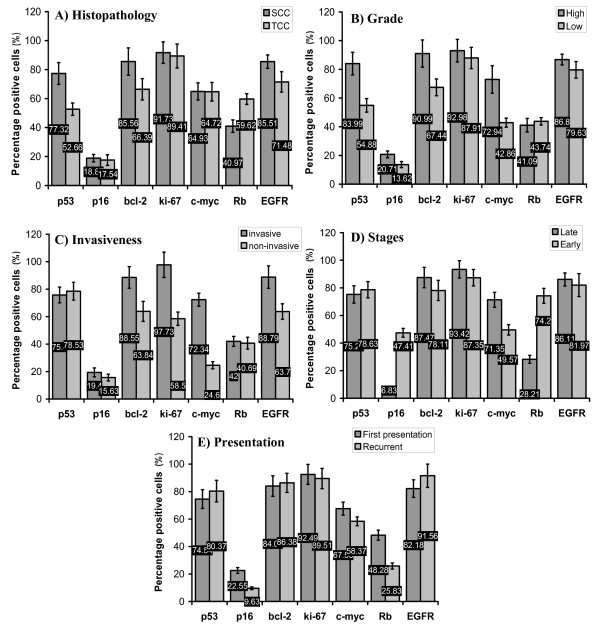
**The mean percentage of the positively immunostained cells for p53, p16, bcl-2, ki-67, c-myc, Rb, and EGFR in tumor tissue sections of SBT in relation to (A) histopathology; SCC and TCC**. (B) Grade of the tumor; high and low grades. (C) Invasiveness of the tumor; invasive and non-invasive (D) Stage of cancer; late (stages VI and III) and early stages (stages I and II). (E) Presentation of the disease; first presentation and recurrent.

Regarding SBT, p53, bcl-2, and EGFR were found higher and Rb lower in SCC than in TCC (P < 0.05) (Figure. 3-A). p53, bcl-2, p16, and c-myc were higher in high grade tumors than low grade (P < 0.05) (Figure. 3-B). Bcl-2, ki-67, c-myc, and EGFR were associated with invasive tumors and the highest association was found in c-myc (P < 0.05) (Figure. 3-C). P16 and Rb were severely lowered in late stages of the disease (III and IV) while c-myc was increased (P < 0.05) (Figure. 3-D). It was also found that Rb and p16 were lowered in the recurrent presentation while c-myc was higher in the first presentation (P < 0.05) (Figure. 3-E).

**Figure 4:**
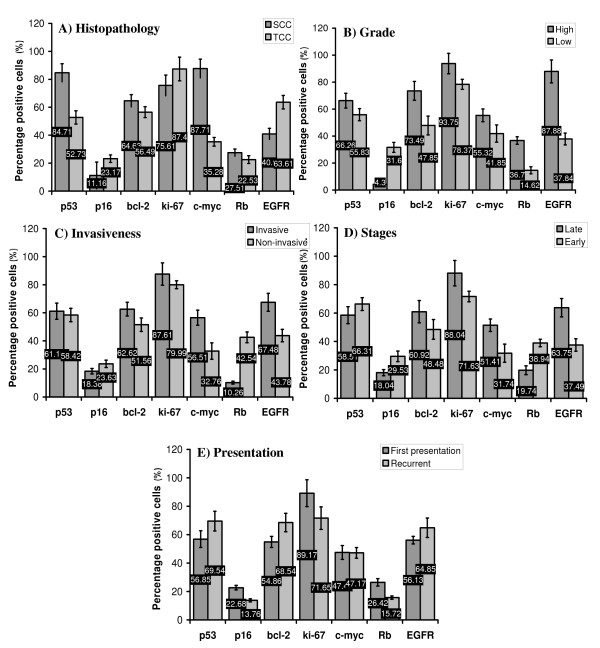
**The mean percentage of the positively immunostained cells for p53, p16, bcl-2, ki-67, c-myc, Rb, and EGFR in tumor tissue sections of NSBT in relation to (A) histopathology; SCC and TCC**. (B) Grade of the tumor; high and low grades. (C) Invasiveness of the tumor; invasive and non-invasive. (D) Stage of cancer; late (stages VI and III) and early stages (stages I and II). (E) Presentation of the disease; first presentation and recurrent.

Regarding NSBT, only p53 and c-myc were clearly associated with SCC while EGFR, unlike in SBT, was associated with TCC (P < 0.05) (Figure. 4-A). All studied markers were higher in high grade tumors than in low grade and p16 was very low in high grade tumors (P < 0.05) (Figure. 4-B). Bcl-2, c-myc, and EGFR were higher in invasive than in non-invasive tumors while p16 and Rb, unlike in SBT, were lower in invasive than in non-invasive (P < 0.05) (Figure. 4-C). Ki-67, c-myc, and EGFR were higher in late stages of the disease than early stages while p16 and Rb were lower in late than early stages (P < 0.05) (Figure. 4-D). Bcl-2 was higher and p16 and Rb were lower in recurrent than in first presentation (P < 0.05) (Figure. 4-E).

The behavior of the studied markers in SBT and NSBT was sometimes similar and sometimes different in relation to the clinicopathological criteria. Collectively, in both SBT and NSBT, the similar behavior of the studied markers was as follows; a) p53 was associated with SCC. b) p53, bcl-2, and c-myc were higher in high grade tumors. c) Bcl-2, c-myc, and EGFR were higher in invasive than non-invasive tumors. d) P16 and Rb were lowered in late stages of the disease (III and IV) while c-myc was higher. e) Rb and p16 were lowered in the recurrent presentation.

On the other hand, the main lines of difference in the expression of the studied markers between SBT and NSBT were briefly as follows: a) In SBT, bcl-2, Rb, and EGFR were associated with SCC while in NSBT c-myc was associated with SCC and EGFR was associated with TCC. b) ki-67, Rb, and EGFR were higher in high grade tumors in NSBT rather than SBT. c) ki-67 was higher in invasive than in non-invasive tumors in SBT while p16 and Rb were lower in invasive than in non-invasive in NSBT. d) EGFR and ki-67 were higher in late stages of the disease in NSBT only. e) Bcl-2 in NSBT was higher in recurrent cases than first time presentation.

### Correlation coefficients among the studied markers

The Pearson's correlation coefficient (*r*) was calculated to measure the correlating behavior among the different studied markers in SBT and NSBT. Briefly, it was found that c-myc in both SBT and NSBT was inversely correlated with p16, r = -0.74 and r = -0.68 respectively, and Rb, r = -0.83 and r = -0.89 respectively (P < 0.05). p53 was positively correlated with bcl-2, r = +0.72, in SBT (P < 0.05) but not in NSBT. EGFR was positively correlated with c-myc in both SBT, r = +0.57, and NSBT, r = +0.61 (P < 0.05). And p16 was inversely correlated with p53 in SBT, r = -0.59, and NSBT, r = -0.64 (P < 0.05).

## Discussion

This study confirmed that the Middle East is greatly affected by schistomiasis. In this study, SBT was 53.57% of the involved cases of bladder cancer. In addition, the mean age of SC and SBT patients was lower than in NSC and NSBT respectively with significant male predominance in SBT and SC cases. This indicated that schistomal infection speeds up the incidence of SC and SBT. This finding was supported by another report which revealed that the development of SBT occurs in younger age group, 49.4 years [7] and 51.4 years [19] where it affects males predominantly.

SBT was associated significantly with SCC, high grade, and invasive tumors while NSBT was associated with TCC, a bit lower grade, and less invasive tumors. This provided evidence that the molecular basis and the underlying mechanisms of cancer development in SBT and NSBT might be different. Regarding the association of SBT with SCC, this study was congruous with other reports [6,19] but this study showed that SBT is associated more with high grade tumors and disagreed with other studies [19,20] conducted in Egypt which revealed that SBT is associated more with low grade tumors. Unfortunately no studies were conducted in the same region of our study in order to compare. Nevertheless, the possible explanation of this variation might be attributed to the geographical variation between the Nile river valley in Egypt and that in Jordan, Syria and Iraq.

Alterations in cell cycle, oncogenic, and apoptotic proteins are the key events in determining the biological behavior of bladder cancer [21]. This study provided evidence that the biological behavior between SBT and NSBT and between SC/NSC and CTL groups was different. However, no remarkable differences were found between SC and NSC groups. The expression level of the all studied markers, except for p16 and ki-67 proteins, was different between SBT and NSBT. p53, bcl-2, c-myc, Rb, and EGFR proteins were significantly higher in SBT than in NSBT. This could highlight the important targets of anticancer therapy in SBT and NSBT. Surprisingly, the cystitis patients, who were confirmed free of any premalignant lesions, showed higher expression of p53, bcl-2, ki-67, and EGFR but not c-myc, p16, and Rb proteins than in CTL group. This provided a clue that both SC and NSC might act as an intermediate stage between normal and tumorous tissues indicating the danger of the long-lasing inflammation of the bladder. Chronic cystitis appeared as transitional phase for increasing cells turnover (ki-67), decreasing tumor suppression capability (p53), decreasing the apoptotic potential (bcl-2), and increasing the cells' response to growth stimuli (EGFR) which all prepare the suitable background of malignancy. On the other hand, c-myc and Rb did not appear much affected during the chronic cystitis phase.

The expression of p53 protein was higher in SBT than in NSBT, higher in NSBT than in SC/NSC, and higher in SC/NSC than in CTL. It was highly expressed in high grade SCC in both SBT and NSBT. Therefore, p53 could be exploited as a useful indicator for high grade SCC bladder cancer in general and in SBT in particular. These results are in agreement with other reports which showed that 72% [22] and 73% [23] of the SBT cases expressed immunoreactive p53. In addition, the current study showed that the higher the p53, the higher the grade of tumor. This is in agreement with other reports showing that p53 was detected in 75% of high grade bladder tumor and 25% of low grade tumors [24] and p53 expression is higher in the poorly differentiated SBT tumors [25]. The current study did not show any association of p53 with disease staging and presentation. This indicates that p53 is not a reliable prognostic factor for both SBT and NSBT. This finding was supported by a study [26] which stated that no evidence has proved the reliability of p53 as prognostic factor in bladder cancer. However, another report stated that p53 is an independent prognostic factor in SCC and TCC bladder cancer [27].

Regarding p16, there was no difference in the expression of p16 between SBT and NSBT but it was remarkably lower in both SBT and NSBT than in SC, NSC, and CTL groups. However, it was stated that p16 genes were altered and deleted in schistosomal bladder cancer [12,28]. Unlike p53, p16 appeared as a reliable marker for assessing the grade and invasiveness of NSBT rather than SBT. In addition, p16 appeared to serve as a good prognostic factor in both SBT and NSBT. This study revealed clearly the association of p16 with disease staging and presentation which was strongly supported by another report [29]. This study also showed that p16 is inversely correlated with p53 indicating that the more mutated p53, the more overexpression of dysfunctional p53, the less p16 proteins will be transcribed.

Rb expression was severely diminished in NSBT and SBT when compared to SC/NSC and CTL groups and was significantly lower in NSBT than in SBT. In addition, Rb was associated with SCC SBT, invasive NSBT, and late and recurrent SBT and NSBT. Therefore, Rb protein can be used as an efficient prognostic and discriminatory factor for both SBT and NSBT. This might give a clue that schistosomiasis has no particular relationship with *Rb *gene in bladder cancer. There is a report [30] revealed that infrequent loss of Rb expression was found in invasive lesions associated with schistosomiasis. Unfortunately, no previous study was done on the association of Rb with SBT in the same geographical region of the current study. Interestingly, both Rb and p16 proteins were inversely correlated with c-myc in both SBT and NSBT. A recent study [31] found that the mechanism of Rb inactivation is through hyperphosphorylation, which results from loss of p16 expression.

Bcl-2 protein was similar to that of p53. It was higher in SBT than in NSBT, in SBT/NSBT than in SC/NSC, and in SC/NSC than CTL. And it was associated with SCC SBT and high grade invasive SBT and NSBT. Moreover, it was not associated with staging, presentation or TCC NSBT. Accordingly, bcl-2 proved to be a useful discriminatory factor between SBT and NSBT, cystitis and bladder cancer, and cancer/cystitis and CTL. This study showed that bcl-2, or loss of apoptotic potential, increases steadily with bladder chronic inflammation and with bladder cancer favoring SBT on NSBT. These findings are in agreement with [24] who stated that the positive immunostaining of bcl-2 was observed in 69% of bladder cancers where 75% of patients were with high-grade tumors. In addition, the current study supports a recent report [32] stating that bcl-2 is of little prognostic value. However, our findings contradict another report [23] which showed that bcl-2 expression was only 20% in schistosomal bladder cancer and it has no relationship with tumor grade. On the other hand, the current study confirmed the presence of significant direct correlation between bcl-2 and p53 which supports the conclusions of another report [16] stating that the loss of p53 function enhances the expression of bcl-2, by relieving it from the transcriptional repression of the wild type p53 protein.

Regarding oncogenes, c-myc was higher in SBT than in NSBT, higher in SBT/NSBT than in other groups. It was associated with tumor grade, invasiveness, and late stages in both SBT and NSBT. It was the only factor associated with tumor invasiveness, grade, and prognosis as well as it proved to be a good discriminatory factor between SBT and NSBT and between bladder cancer and cystitis/CTL groups. These findings are in agreement with [33] who showed that 58% of bladder cancer patients were c-myc positive and 59% of the positive cases were of muscle-invasive tumors. However unlike the results of our study, they concluded that c-myc over-expression did not correlate with tumor grade or tumor progression while another study [34] found that 34% of patients had positive c-myc which was associated with tumor grade but with no prognostic value. Unfortunately, no previous study was conducted on the association of c-myc with SBT to compare with. The current study might be the first to investigate the role of c-myc in SBT and NSBT and might be the first to relate c-myc with the clinicopathological criteria of bladder cancer.

Regarding EGFR, this oncogene increased significantly from CTL towards NSC, SC, NSBT, and SBT. Therefore EGFR could be used as a reliable discriminatory factor for the all studied groups. Moreover, it was associated with SBT SCC, NSBT TCC, high grade NSBT, invasive SBT and NSBT, and late stages of NSBT. The results of the current study are supported by a study [35] which stated that EGFR is associated with SCC of bladder and another study [10] stated that 70% of muscle-invasive bladder cancers express EGFR which is associated with poor prognosis. Accordingly, the current study showed the importance of EGFR as a candidate for anti-cancer therapy in bladder. It was suggested that there is a need to use anti-EGFR as a novel anti-cancer therapy in bladder [11].

In cancer cells, Ki-67 plays an important role as an index for the replication and the prognosis and is well associated to tumor grade, stage and recurrence [36]. In this study, expression of Ki-67 protein was higher in SBT/NSBT than in SC/NSC which was higher than in CTL group. There was no difference in the proliferation rate between SBT and NSBT. Therefore, a limited role of ki-67 might be present in schistosoma-related pathogenesis of bladder cancers. Moreover, ki-67 was associated with high grade NSBT, invasive SBT, and late stage NSBT. This is in agreement with other studies [37,38] which showed that Ki-67 positive immunostaining was correlated with tumor grade and muscle invasion.

## Conclusion

Taken together, the molecular background of SBT seems distinct from that of NSBT. SBT was associated with SCC, higher grade and more invasive tumors while NSBT was associated with TCC, lower grade and less invasive tumors. p53, bcl-2, c-myc, Rb, and EGFR were highly expressed in SBT, more than in NSBT, which are therefore might be useful as indicators and discriminatory markers for bladder cancer in general and SBT in particular. Chronic cystitis acts as an intermediate stage for the overexpression of p53, bcl-2, and EGFR markers that were shown implicated in both SBT and NSBT. p53 is strongly associated with high grade SCC tumors in both SBT and NSBT but it is poor prognostic factor. Bcl-2 is similar to p53 but it is increased in recurrent cases. P16, Rb, and c-myc were shown as good prognostic markers for SBT and NSBT. C-myc and EGFR appeared central in many aspects of carcinogenesis, tumor grade, tumor invasiveness, and cancer staging.

## Competing interests

The authors declare that they have no competing interests.

## Authors' contributions

RR and HS carried out patients sampling and interviewing in conjunction with specialist urologists. AS and F did the immunostaining procedures and examination in conjunction with specialist pathologists. AS and F carried out the paper drafting, statistical design, statistical analysis, and the proofreading of the article language and integrity. All authors read and approved the final manuscript.
